# Whispering to the Deaf: Communication by a Frog without External Vocal Sac or Tympanum in Noisy Environments

**DOI:** 10.1371/journal.pone.0022080

**Published:** 2011-07-13

**Authors:** Renaud Boistel, Thierry Aubin, Peter Cloetens, Max Langer, Brigitte Gillet, Patrice Josset, Nicolas Pollet, Anthony Herrel

**Affiliations:** 1 Centre de Neurosciences Paris-Sud (CNPS), Centre National de la Recherche Scientifique (CNRS), UMR8195, Université Paris XI, Orsay, France; 2 Institut International de Paléoprimatologie et de Paléontologie Humaine (IPHEP), Centre National de la Recherche Scientifique (CNRS), UMR 6046, Université de Poitiers, Poitiers, France; 3 European Synchrotron Radiation Facility, Grenoble, France; 4 Centre de Recherche en Acquisition et Traitement de l'Image pour la Santé (CREATIS), Centre National de la Recherche Scientifique (CNRS), UMR 5220, Institut National de la Santé et de la Recherche Médicale (INSERM), U630, Université Claude Bernard Lyon 1, Institut National des Sciences Appliquées Lyon, Villeurbanne, France; 5 Imagerie par Résonance Magnétique Médicale et Multi-Modalités (IR4M), Centre National de la Recherche Scientifique (CNRS), UMR8081, Université Paris-Sud, Orsay, France; 6 Hôpital d'Enfants Armand Trousseau, Paris, France; 7 Institute of Systems and Synthetic Biology, Genopole, Centre National de la Recherche Scientifique (CNRS), University of Evry, Evry, France; 8 Département Ecologie et Gestion de la Biodiversité (EGB), Centre National de la Recherche Scientifique (CNRS), Muséum National d'Histoire Naturelle, UMR 7179, Paris, France; University of Auckland, New Zealand

## Abstract

*Atelopus franciscus* is a diurnal bufonid frog that lives in South-American tropical rain forests. As in many other frogs, males produce calls to defend their territories and attract females. However, this species is a so-called “earless” frog lacking an external tympanum and is thus anatomically deaf. Moreover, *A. franciscus* has no external vocal sac and lives in a sound constraining environment along river banks where it competes with other calling frogs. Despite these constraints, male *A. franciscus* reply acoustically to the calls of conspecifics in the field. To resolve this apparent paradox, we studied the vocal apparatus and middle-ear, analysed signal content of the calls, examined sound and signal content propagation in its natural habitat, and performed playback experiments. We show that *A. franciscus* males can produce only low intensity calls that propagate a short distance (<8 m) as a result of the lack of an external vocal sac. The species-specific coding of the signal is based on the pulse duration, providing a simple coding that is efficient as it allows discrimination from calls of sympatric frogs. Moreover, the signal is redundant and consequently adapted to noisy environments. As such a coding system can be efficient only at short-range, territory holders established themselves at short distances from each other. Finally, we show that the middle-ear of *A. franciscus* does not present any particular adaptations to compensate for the lack of an external tympanum, suggesting the existence of extra-tympanic pathways for sound propagation.

## Introduction

Vocalizations play an important role in the mating and territorial behavior of most anurans [1]. The most obvious acoustic signals produced by these vertebrates are the advertisement calls [2] emitted during the breeding season. *Atelopus* is a large genus (80 species) of diurnal bufonids (known as “Harlequin frogs”) which is remarkably uniform in morphology [3] and call structure [3], [4]. Visual signals during agonistic displays between males are common [5] and involve movements of the forelimbs. The use of these particular visual signals in the genus *Atelopus* is intriguing because these frogs also vocalize [5]. Interestingly, and in contrast to other species of the genus, *Atelopus franciscus* males only use advertisement calls and no visual displays to attract females and defend territories [6]. This is unexpected, however, given that they lack an external vocal sac, and that they live in a noisy calling environment consisting of river banks in tropical forest. Indeed, without an external vocal sac, *A. franciscus* likely exhibits a low energetic efficiency for calling, since the range over which acoustic signals propagate depends on the power generated by the sound source. In fact, less than 0.05% of the source (larynx) energy is susceptible to transfer into the medium [7], and the solution observed in most frogs is the coupling of the larynx (source) to a resonator (the external vocal sac) tuned to the frequency of the signal. Moreover, the structure of the advertisement call appears poorly adapted to a noisy environment. Contrary to forest stream dwelling Asian frogs [8], [9], [10], *A. franciscus* produces multi-pulsed units separated by short silences [11], typical of species living in open environments (ponds and paddy fields, [8]). The emission of advertisement calls in an absorbent and noisy environment imposes limits on the ability to communicate as calls suffer attenuation and degradation. Communication in this habitat is rendered even more difficult by the presence of numerous other frog species producing high energy calls.

The last, and perhaps the most intriguing aspect of communication in this frog, is related to the ear anatomy of *A. franciscus*. In contrast to other amphibians (salamanders and caecilians), the majority of anurans have developed tympanic middle ears, consisting of an external tympanum, a middle ear cavity, an eustachian tube, and auditory ossicles [12], [13]. However, most *Atelopus*, including *A. franciscus*, lack an external tympanum [13]. Without the external tympanum acting as an interface between the external environment and the inner ear, this species can be considered anatomically deaf. Paradoxically, *Atelopus* species have standard inner ears with well-developed auditory sensory organs [12], and neurophysiological studies have established that they have the same sensitivity to sound as species with an external tympanum [12], [14].

As acoustic communication is dependent on efficient emission, propagation and reception of a signal, the system fails if one of these elements is disrupted. The aim of this study was to examine the adaptations that allow this frog without an external tympanum and vocal sac to transmit information in an absorbent and noisy environment. To do so, we studied its acoustic communication system at the different levels of the information transmission chain. First, we studied the encoding of information by analysing the physical properties of the vocal apparatus and the acoustic structure of the advertisement call. Second, we explored signal modifications occurring during transmission from the emitter to the receiver. Third, we examined the anatomy of the middle-ear using histology, synchrotron X-ray phase contrast microtomography, and magnetic resonance imaging (MRI). Finally, using field-based playback experiments with modified signals, we deciphered the coding system that allows species-specific recognition in this frog.

## Results

### Vocal apparatus and call intensity

**Figure 1:**
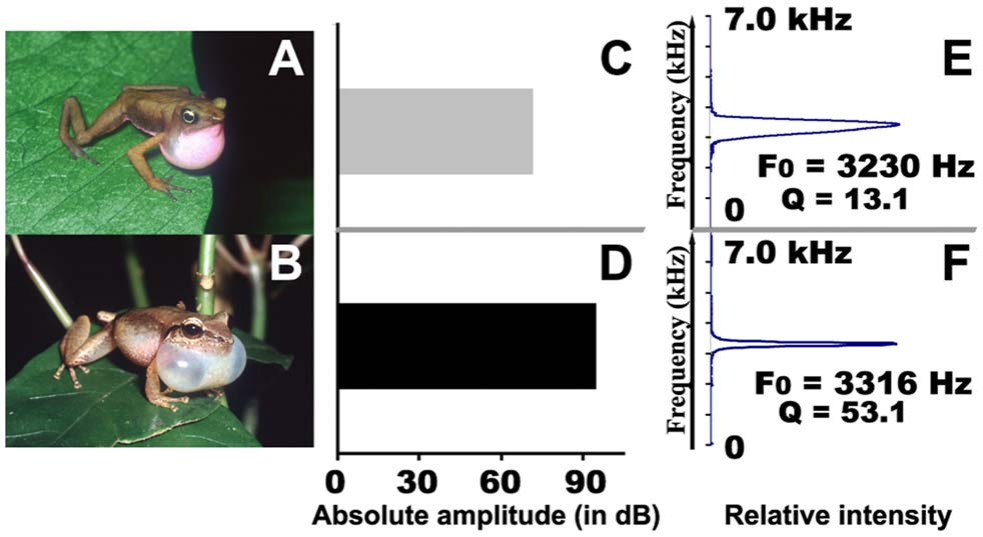
Comparison of the vocal performance of two anurans emitting calls at a similar frequency. (a) *Atelopus franciscus*, which has an internal vocal sac, and (b) *Eleutherodactylus martinicensis*, which has an external vocal sac. The emission intensity (c, d) and the resonant frequency and Q factor (e, f) are shown for both species. Note how the two species have differences in the relative resonator bandwidth. In *A. franciscus* the bandwidth is wider and the call is of low intensity as a consequence of its internal vocal sac.

The nomenclature of the vocal sac used here is based on Liu [15]. For comparative purposes we compared call quality factor (Q) and sound pressure levels (SPL) of *A. franciscus* with those of a species emitting a signal with a similar dominant frequency, *Eleutherodactylus martinicensis* (Fig. 1). Whereas *A. franciscus* has an internal vocal sac with fine and long vocal slits, *E. martinicensis* has an external vocal sac. The mean Q values of *A. franciscus* and *E. martinicensis* calls were 13.11 (*n* = 142) and 53.1 (*n* = 18) respectively. The mean (± SD) SPL values of *A. franciscus* and *E. martinicensis* were 72±3.4 dB (*n* = 11) and 95±3.9 dB (*n* = 6) respectively.

### Ear anatomy (Figure 2, Video S1, Video S2)

**Figure 2:**
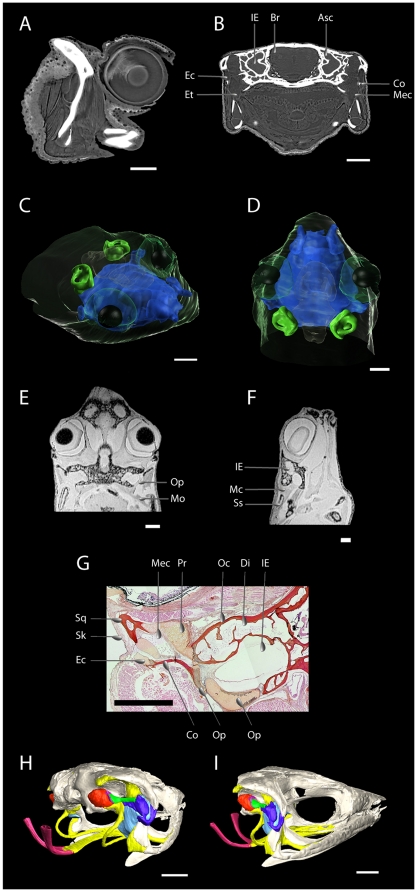
Ear anatomy of *Atelopus franciscus*. (a) View of the pseudo-tympanum (note that the slices are windowed to obtain the best contrast in soft tissues) consisting of the extra-columella and middle ear cavity on a virtual medial slice of the left ear obtained with holotomography (7.46 µm). (b) A virtual coronal slice at the level of pseudo-tympanum showing the connection of the middle ear with the buccal cavity obtained with holotomography (7.46 µm). (c) and (d) 3D visualisation of inner ear (green) and middle ear cavity connected to the buccal cavity (blue) with the skin rendered transparent. (e) and (f) Views of the connection between the opercular muscle, the suprascapula, and the operculum. The virtual slices are obtained by MRI in the saggital and frontal plane. (g) Histological section of the left ear in the frontal plane illustrating the fibrous connection between the middle ear ossicle and the otic capsule. (h) and (i) Volume rendering of middle ear anatomy inside the skull obtained with holotomography (10 µm). (h) lateral view, (i) postero-lateral view. Scale bars represent 1 mm. Abbreviations: Asc Anterior semi-circular canal, Br Brain, Co columella, Di diploe, Ec extracolumella, Et Eustachian tubes, IE inner ear, Ip interna plectri, Mec middle ear cavity, Mo muscle opercular, Oc otic capsule, Op operculum, Pr prootic, Sk Skin, Sq squamosal and Ss suprascapula.

The nomenclature used here to describe the ear is based on Wever [12]. In contrast to most other anurans, *A. franciscus* lacks a tympanic membrane and a tympanic annulus. The area corresponding to the tympanic membrane in other frogs is not differentiated from the surrounding skin and shows skin glands. Under the skin, a pseudo-tympanum (PT) is present which is, however, smaller (area: 0.34 mm^2^) than the oval window of other ranoid species. The columella is well developed and consists of three plectral parts. The median plectrum (length: 1.26 mm) runs horizontally along the dorsal wall of the middle ear cavity. The cartilaginous extracolumella (length: 0.73 mm) is different from that of ranids [16], [17] and is attached by its slim distal end to a large cartilage cushion occupying the superior part of the inner aspect of the pseudo tympanum. The extra-columella is attached to the skull by means of a strap-like cartilaginous process (the ascending process). A short but wide eustachian tube connects the middle ear with the posterior corner of the buccal cavity. A lateral opening in the inner-ear capsule, the oval window (surface: 0.68 mm^2^), is covered by a convex external and concave internal operculum and a columellar footplate (length: 0.76 mm, contact area with the oval window: 0.15 mm^2^). These elements are separated and connect without interdigitation. The surface ratio is lower, and the impedance transformer ratio (ITR) of the middle ear is higher, than those reported for other tetrapods (Table 1).

**Table 1:** Comparison of the lever ratio, ITR and transmission of the middle ear in tetrapods.

Vertebrate	Animal	Ratio T/FO	Ratio lever ossicle	ITR	Tmax (in %)	Tmin (in %)	Source
Anuran	*Atelopus franciscus*	0.5	3.3	0.183	14.99	7.79	current study
	*Bufo americanus*	12.8	/				[S1]
	*Hyla cinerea*	9.1	/				[S1]
	*Rana catesbeiana*	27–47	1.2	0.044–0.0161	86.3	60.8	[S2]
	*Rana temporaria*	/	5.8				[S3]
Mammal	*Human*	17	1.2	0.041	51.99	30.5	[S4]
	*Primate*	21.1	1.8	0.014	89.94	65.39	[S5]
	*Cat*	/	2				[S6]
	*Bats with sonar*	16	2.3	0.012	95.26	73.61	S7]
	*Bats without sonar*	14.7	1.7	0.023	73.8	47.9	[S7]
Bird	*Pigeon*	21	2.7	0.007	99.6	92.38	[S8, S9]
	*Chicken*	11	/				[S10]
	*Grebes (Podiceps)*	15	/				[S10]
	*Birds of prey (falconiformes)*	20	/				[S10]
	*Owls (strigiformes)*	30	1.6	0.013	92.46	68.98	[S11]
Lizard	*Gekkonidae*	28.6	4.3	0.002	64.51	89.28	[S12]

**References**

S1. Moffat AJM, Capranica RR (1978) Middle ear sensitivity in anurans and reptiles measured by light scattering spectroscopy. J Comp Physiol 187 [A]: 97-107.

S2. Mason MJ, Narins PM (2002) Vibrometric studies of the middle ear of the bullfrog Rana catesbeiana I, The extrastapes. J Exp Biol 205:3153-3165.

S3. Jørgensen MB, Kanneworff M (1998) Middle ear transmission in the grass frog, *Rana temporaria*. J Comp Physiol [A]. 182: 59-64.

S4. David R (2002) Signals and Perception: The Fundamentals of Human Sensation. Basingstoke: Palgrave, Open University. 407.

S5. Coleman MN, Ross CF (2004) Primate auditory diversity and its influence on hearing performance. Anat Rec 281A: 1123-1137.

S6. Iskandar HG, IH,Mounir M (1982) The ossicular system of cats. J Laryngol Otol 96: 195-204.

S7. Thomassen HA, Gea S, Maas S, Bout RG, Dirckx JJJ, Decraemer WF, Povel GDE (2007) Do Swiftlets have an ear for echolocation? The functional morphology of Swiftlets middle ears. Hear Res 225: 25-37.

S8. Schwartzkopff J (1955) On the hearing of birds. Auk 72: 340-347.

S9. Gummer AW, Smolders JWTh, Klinke R (1989) Mechanics of a single-ossicle ear: I. The extra-stapedius of the pigeon. Hear Res 39: 1-13.

S10. Saunders JC, Duncan RK, Doan DE, Werner YL (2000) The middle ear of reptiles and birds. In: Comparative Hearing: Dooling RJ, Fay RR, Popper AN, editors. Birds and Reptiles, New York: Springer-Verlag. 13-69.

S11. Payne RS (1971) Acoustic Location of Prey by Barn Owls (*Tyto Alba*). J Exp Biol 54: 535-573.

S12. WernerYL, Igić PG (2002) The middle ear of gekkonoid lizards: interspecific variation of structure in relation to body size and to auditory sensitivity. Hear Res 167:33-45.

### Call analysis (Figure 3, Audio S1)

**Figure 3:**
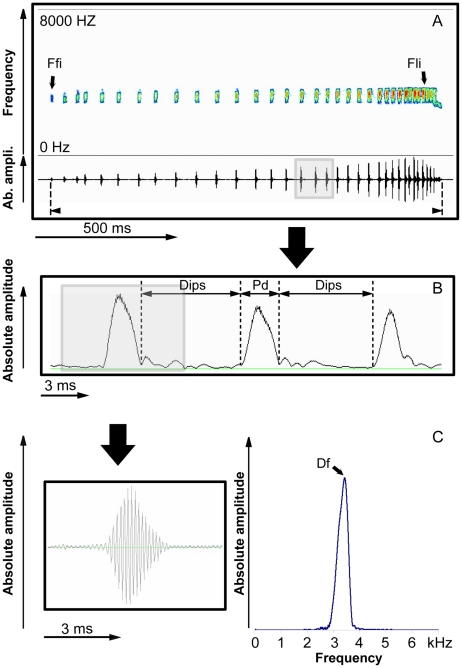
Advertisement call of *Atelopus francisus*. (a) sonographic and oscillographic representations of a natural call, (b) envelope representation of three successive pulses corresponding to the part highlighted in (a), c) oscillographic (left) and spectrographic (right) representation of a single pulse. Abbreviations refer to temporal and frequency measures.

Call variability was assessed by analyzing 130 advertisement calls from 14 males (Table 2). The mean call duration was 1.33±0.25 s. The call is emitted at a rate of 15 calls/min separated by silences of 3.26±1.83 s. The signal consists of 32±4.5 pulses of 3.62±0.93 ms each, emitted at a rate of 25.03±6.10 pulses/s, and separated by silences of 39.11±21. 93 ms. Call frequency increased with the increase in amplitude from the beginning to the end of the signal and reached a maximum value of 800 Hz. Spectral analysis of pulses by FFT shows that the call consists of a pure sound with a frequency at a mean value of 3134±187 Hz. Two parameters of the call (pulse duration, Pd and the dominant frequency, Df) appear highly repeatable and stereotyped and can be considered as species-specific characteristics.

**Table 2:** Acoustic parameters and coefficients of variation (C.V.) measured on the advertisement calls of 14 individuals (with a mean number of 10 calls per individual).

Parameters	Avg.	S.D.	Min.	Max.	N	CV	aCVi	CVP/aCVi
Sd (s)	38.6	9.63	20.23	55.48	14	\	\	\
Cr	0.25	0.04	0.18	0.33	14	\	\	\
Np/C	32.01	4.49	24	46	129	14.1	7.3	2.4
Cd (s)	1.33	0.25	0.9	1.86	130	18.7	5.8	3.5
Dics (s)	3.26	1.83	1.64	18.26	117	56.5	31.5	2.7
Pd (ms)	3.62	0.93	2	6.94	4120	25.8	23	1.2
Dips (ms)	39.11	21.93	0.25	133.13	3991	56.1	47.4	1.2
Pr	25.03	5.94	15.84	37.75	126	23.9	7.8	3.6
R	0.1	0.03	0.05	0.23	126	33.3	13.8	2.8
FM (Hz)	411	197	0	813	136	48.3	41.6	1.6
Ffi (Hz)	3298	165	2875	3719	136	5	3.7	2
Fli (Hz)	2887	167	2500	3313	136	5.8	3.1	2.2
Df (Hz)	3134	187	2500	3656	1329	6	4.7	1.5

Abbreviations: Avg Average, aCVi average Coefficient of Variation individual, CVP Coefficient of Variation of Population, Max Maximum, Min Minimum, N Number.

### Ambient noise

The ambient noise differed slightly at the two studied sites in the rainforest. We measured 57.09±2.72 dB along the river bank and 52±1.80 dB in the undergrowth (UG). The difference between the two sound pressure levels are statistically significant (Mann-Whitney U test, P<0.001, z = 3.402).

### Call attenuation (Figure 4)

**Figure 4:**
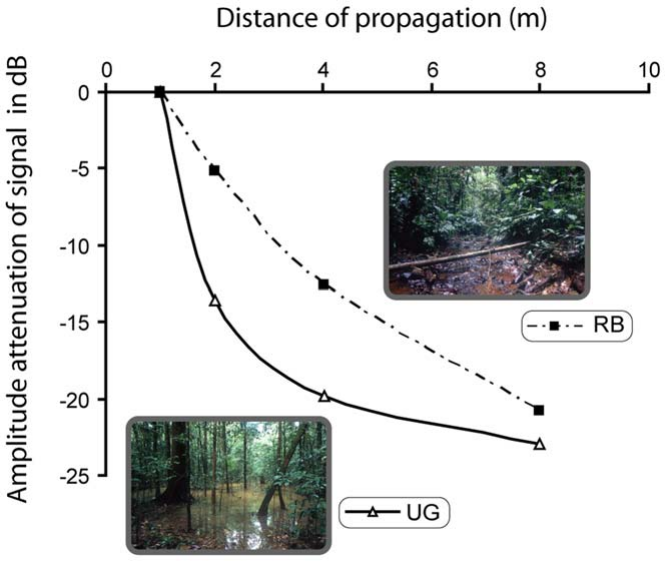
Attenuation of the call propagated at different distances (2, 4, 8 m) in two natural habitats, undergrowth (UG) and riverbank (RB).

The measured attenuation along the riverbank (RB) is well described by the inverse square law for the spreading of spherical waves (-6dB per doubling of distance), similar to what has been described for *Centrolenella fleischmanni* [18]. This is not the case in the undergrowth. Here, a strong decrease of the amplitude (-13.54 dB) was observed over the first two meters, after which the measured attenuation followed the inverse square law. At a distance of 16 m the signal was below the background noise level and attenuation values were not measurable in both sites (riverbank and undergrowth).

### Modification of amplitude modulation

At both sites, modifications of the envelopes of the propagated signal compared to the non-propagated signal were small for signals recorded at 2 m and 4 m (Pearson correlations: river bank: *r* = 0.82 at 2 m and *r* = 0.80 at 4 m, *p*<0.05; undergrowth: *r* = 0.87 at 2 m and *r* = 0.85 at 4 m, *p*<0.05). Modifications of the envelopes are stronger beyond 8 m (riverbank: *r* = 0.64; *p*<0.05; undergrowth: *r* = 0.53, *p*<0.05) and at 16 m modifications were no longer measurable as the signals recorded were softer than the background noise.

### Interspecific acoustic competition with other frogs (Figure 5)

**Figure 5:**
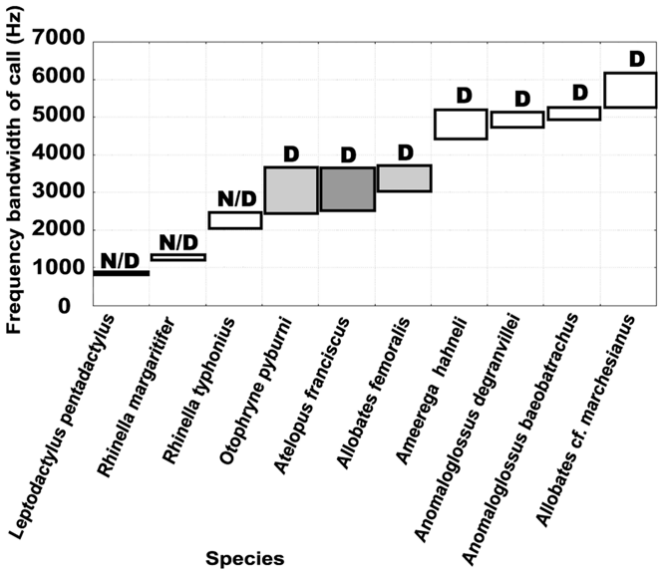
Frequency bandwidth of the calls of 9 frog species sympatric with *A. franciscus*. In light gray, the two species in frequency competition with *A. franciscus*. *A. franciscus* in dark gray. D, diurnal; N/D, diurnal/nocturnal activity.

We identified and recorded 33 species of frogs in our study area. Only 9 of these called during the day and only 2, *Allobates femoralis* and *Otophryne pyburni*, were overlapping in frequency with the call of *A. franciscus*. However, the temporal structure of the calls of these two species differed from that of *A. franciscus*.

### Playback experiments

**Figure 6:**
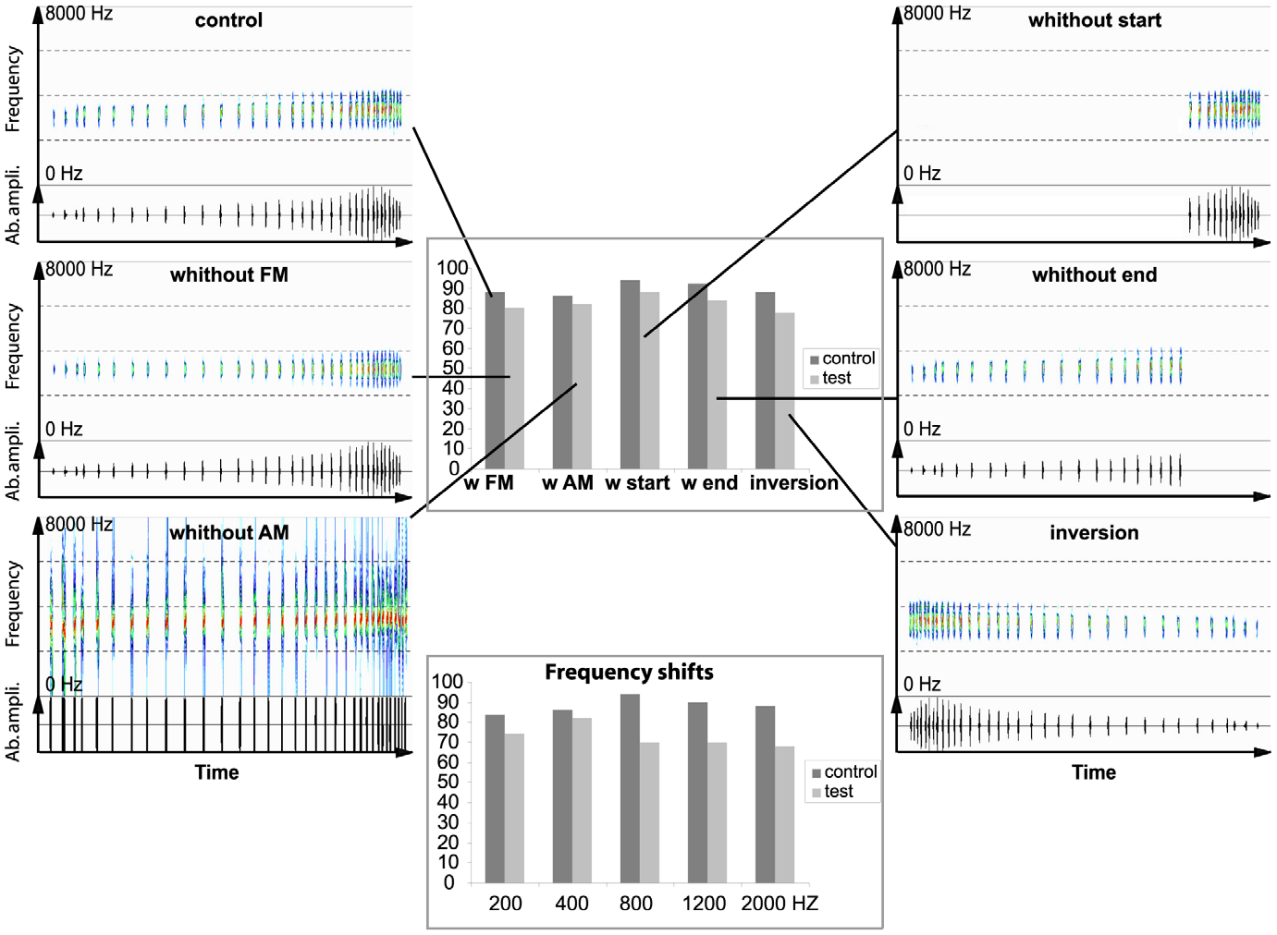
Sonographic and oscillographic representations of test signals used for playback experiments (signals corresponding to tests of frequency parameters are not shown). Histogram representations correspond to the associated responses expressed as a proportion of the theoretically maximal response score.

**Figure 7:**
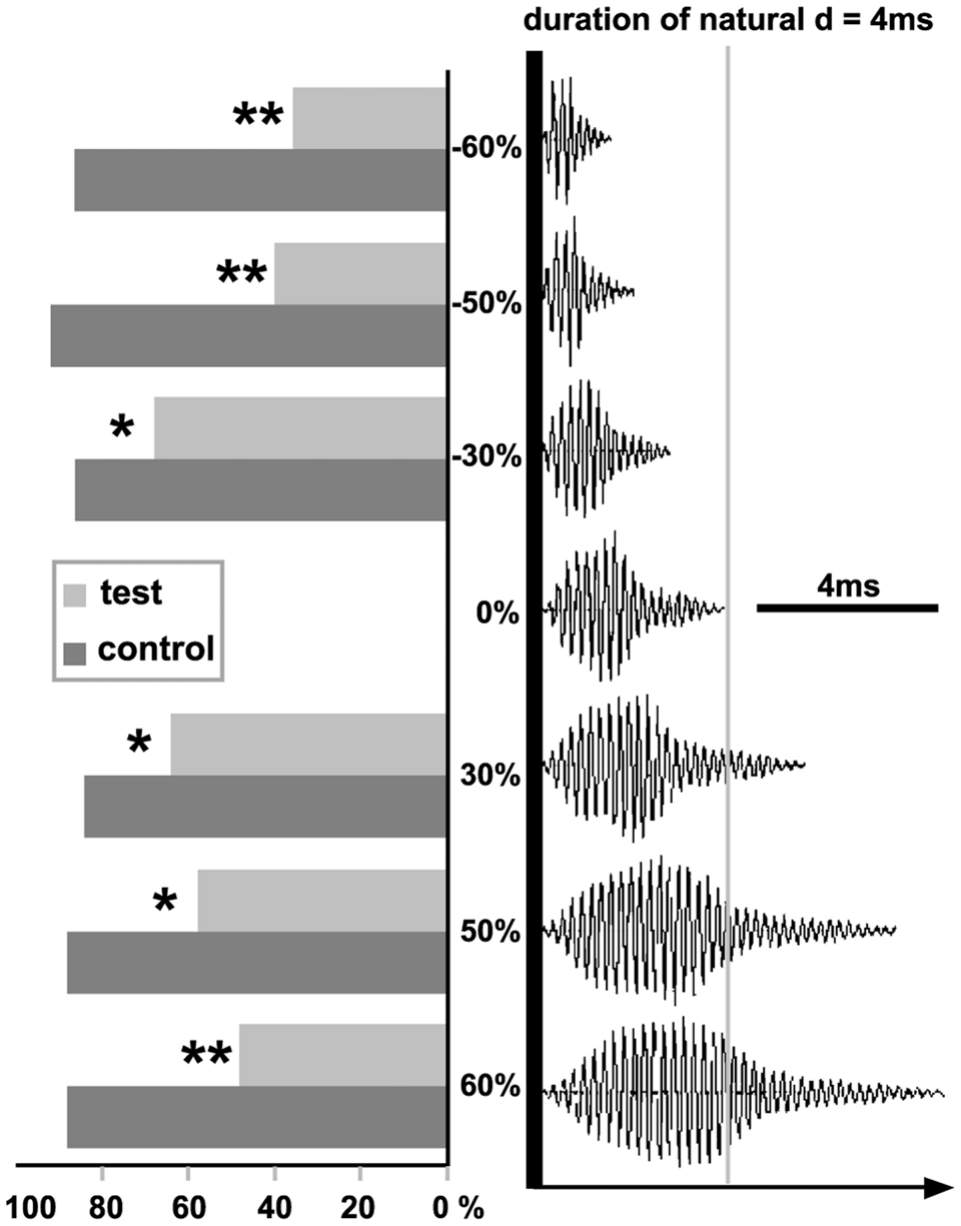
Oscillographic representations of test signals used during playback experiments with natural pulses stretched or compressed (expressed in %). Histogram representations correspond to the associated responses expressed as a proportion of the theoretically maximal response (*  =  p<0.05, **  =  p<0.01).

We did not observe a significant difference between the response to the control signal and the experimental signal where amplitude modulation was suppressed (sign test, *p* = 1, *n* = 10; Fig. 6). Inversion of the temporal structure of the signal also did not elicit a different response (sign test, *p* = 0.13, *n* = 9, Fig. 6). We did not observe a significant difference between the response to the control signal and responses to the signal with only the first part of the call (sign test, *p* = 0.13, *n* = 9, Fig. 6), or only the final part of the call (sign test, *p* = 0.48, *n* = 9, Fig. 6) present. We did not observe a significant difference between the responses to the control signal and the experimental signals with the frequency shifted (sign tests: +200 Hz, *p* = 0.13; +400 Hz, *p* = 0.48; +800 Hz, *p* = 0.22; +200 Hz, *p* = 0.72; +2000 Hz, *p* = 0. 37; n = 10 for all tests, Fig. 6). Finally, We did not observe a significant difference between the response to the control signal and the experimental signal without any frequency modulation (sign test, *p* = 0, 62, *n* = 10, Fig. 6). However, signals with pulse and inter-pulse durations shortened or lengthened elicited significantly weaker responses than the control signal (sign test, *p*<0.05, *n* = 10 in all cases, Fig. 7).

## Discussion

Like most frogs, male *Atelopus franciscus* use advertisement calls to attract females and to defend their territories. Such signals are typically devoted to long-range communication. However, our data show that the Q-value of the call is low. A low Q value is typically associated with short broadband signals that are only slightly longer than the exciting impulse. This is exactly what we observe for the advertisement call of *A. franciscus*: it consists of a train of short pulses (mean duration: 3.6 ms), each pulse corresponding to a non-frequency-modulated sound with a wide frequency bandwidth (ranging from 2.9 to 3.3 kHz). This simple call structure is, however, common among frogs [19], [20] and not specific to earless frog species. In bufonid frogs in particular such a call organisation is common [4], [19]. Interestingly, the call of *A. franciscus* is emitted at a low intensity (72 dB SPL) which is likely due to the lack of an external vocal sac. The external vocal sac has an important effect on the intensity of the signal and on the tuning of the resonant frequencies [20] as it enhances sound radiation by generating standing waves (resonance). *Atelopus franciscus*, having only an internal vocal sac, emits a relatively low intensity call compared to other frogs [21], [22]. In most frogs with sizes similar to *A. francisus* the sound level of calling males measured at 1 m is on the order of 87-113 dB [21], [23]. This range corresponds well to the value (95 dB) we have measured for *E. martinicensis*, a species emitting in the same frequency band and living in the rainforest undergrowth, but possessing an external vocal sac. Due to its weak emission intensity, but also to the absorbent properties of the vegetation and the high background noise the environment (52 dB in the undergrowth and 57 dB along the riverbank), the call of *A. franciscus* propagates only at short range. Our experiments in the natural habitat of this species confirm this and show that at a distance of 8 m the signal is masked by background noise. Moreover, the amplitude modulation of the signal is strongly modified in the natural habitat. Such degradation of amplitude modulation (AM) parameters during propagation, highlighted for other frog species [24], can be critical for species recognition. However, as *A. franciscus* maintains contiguous territories with each male being spaced 2–4 m apart on average, information coded at the level of the amplitude structure can still be effectively transmitted between an emitter and a receiver.

Even though the males call in close proximity to one another, the receivers present a characteristic that seems particularly constraining for effective communication: the lack of an external tympanum. In terrestrial frogs with a complete middle ear, the impedance gap between air and fluid is partially reduced by two anatomical specializations that increase the efficiency of sound transmission: 1) the surface area of the external tympanum is greater than that of the oval window, and 2) the columella has a lever action. Based on our observations, the middle ear anatomy of *A. franciscus* presents some specialisations that could partially compensate for the absence of an external tympanum. The lever ratio was approximately 3.3, i.e. somewhat higher than lever ratios reported for most tetrapods, and hence the middle ear lever probably makes a significant contribution to impedance matching. On the other hand, the middle ear of *A. franciscus* also appears to present some limitations. First, the area of the pseudo-tympanum is smaller than that of the oval window resulting in a poor mechanical pressure transformation and consequently the ITR is higher than that of other tetrapods. Second, no inter-digitations are present between the inner operculum and the columellar footplate and the extra-columella is firmly connected to the squamosal leading to a limited coupling between the two ossicles. Thus, despite its high lever ratio, the middle ear of *A. franciscus* does not seem particularly efficient. Nevertheless *A. franciscus* communicates effectively using airborne sounds [5]. A potential explanation for this paradox may reside in the presence of other acoustic pathways. For low-frequency sounds (<1000 Hz), the opercularis system in the inner ear could serve as an extra-tympanic pathway [14]. For higher frequency sounds, displacements of the body wall overlying the lung could be transmitted to the inner ear by this pathway as well [23], [14]. One other possibility is that sound is transmitted to the inner ear from the sides of the head by conduction through bone or other tissues.

Whatever the acoustic pathway used to reach the inner ear, *A. franciscus* responds to the emission of a conspecific advertisement call by an aggressive call and by orienting itself towards the sound source. In frogs, species-specific coding can be carried either by temporal [25] or frequency-dependent elements [26]. According to our playback experiments, males of *A. franciscus* pay little attention to frequency parameters, since advertisement calls shifted up or down up to 800 Hz still elicited positive responses. Similar results were obtained for signals with AM or FM suppressed or signals with the temporal structure inverted (i.e. AM and FM reversed). On the other hand, signals with the natural intra-pulse frequency but with pulse duration enhanced or shortened by 30, 50, or 60% elicited only weak responses. Thus, the pulse duration of the call appears an important parameter for species-specific recognition.

A coding strategy based on pulse duration could appear inappropriate for a species living in a forest habitat as the reflection of sound waves on the numerous obstacles present (e.g. tree trunks, branches, leaves, etc.) induces reverberating sounds and the resulting echo may increase pulse duration. However, as territorial males establish calling sites in close proximity to one another (2–4 m) the signal remains largely unmodified. In addition, the pulse duration information is repeated often throughout the call and this information redundancy is ideally suited for communication in a noisy environment. A coding based on pulse duration also allows male *A. franciscus* to distinguish conspecific calls from those of the other diurnal frogs, some of which emit in the same frequency range.

To conclude, *A. franciscus* is confronted with several acoustic communication problems: a lack of external vocal sac and an external tympanum, a call emitted at a low intensity level, a noisy and absorbent environment and an important inter-specific competition. To solve these problems, the species adopts two kinds of strategies: 1) behavioural strategies involving the use of contiguous territories with males established in close proximity to one another; 2) acoustic strategies involving the use of redundant and simple coding based on pulse duration, and possibly the use of an extra middle-ear pathway to compensate the lack of external tympanum.

## Materials and Methods

### Study site

Harlequin frogs were studied at two locations in French Guiana: between March and mid-May at the Muséum National d'Histoire Naturelle (MNHN) field station of Saint-Eugène (4°51'N; 53°3'W, 65 m elevation) and between May and June, at the station of Nouragues (4°5' N, 52°41' W, 110 m elevation). Recording and playback experiments were done during the active calling period of males (i.e. between 6.00 a.m. and 6.30 p.m.).

### Electro-acoustic material

Calls were recorded at 1 m distance using an omnidirectional Brüel and Kjær serial 4053 microphone connected to a Sony TCD-D100 digital audio tape (DAT) recorder (sampling rate = 48 kHz, frequency response flat within the range 20–20 000 Hz). Signals were A–D converted (16 kHz), stored on a PC, and modified with Syntana signal processing software [27]. Sound pressure level (SPL in dB) measurements were taken using a Bioblock Scientific Sound Level Meter type 5017 (linear frequency scale, Fast setting). For propagation tests, signals were played back by an Aiwa HDS 1000 DAT or a Sony WM6 recorder (frequency range 30–20000 Hz ±1dB) connected to a 12 cm diameter Klein-Hummel Mini-Monitor 201 loudspeaker (frequency response 80–16000 Hz ±1.5 dB) and re-recorded as mentioned above.

### Vocal apparatus

To characterise the resonance properties of the vocal apparatus we measured the resonant frequency and the Q-value or quality factor. The quality factor is a measure of the sharpness of tuning or of the resistive damping of a vibration [28] and allows an assessment of the quality of oscillators and resonators. An estimation of Q for the vocal apparatus is provided by the equation: Q = f0/BW-3dB SPL [29], where f0 is the resonant frequency (the frequency at which the amplitude is maximal), BW-3dB SPL is the bandwidth at the 50% power level which is at -3dB SPL (20 mPa below the peak), i.e. the frequency range that is within 3dB of the maximum peak amplitude.

### Middle ear biomechanics

The two primary mechanisms that have been proposed to assist in overcoming the impedance mismatch between the perilymph and air [30] are the ossicular lever [31] (the lever action that results from taking half the length of the footplate as the “effective” length of the in-lever and the distance from the fulcrum to the centre of the eardrum of the out-lever) and the areal convergence ratio (i.e. the force collected by the larger surface area of the tympanic membrane is concentrated on the smaller area of the oval window resulting in a pressure amplification; Fig. 2). Using these two ratios we calculated the impedance transformer ratio (ITR) of the middle ear, using the equation provided in Coleman and Ross [32]. Using this ITR, we estimated the theoretical maximum percentage of acoustic transmission (T) at peak performance through the middle ear.

### Call intensity

To characterize the intensity of the call produced by the species, we measured the Sound Pressure Level (SPL) on 11 individuals. The sound level meter was positioned at 1 m in front of the head of male *A. franciscus*. For each individual, 10 measures were taken at 15 s intervals. All these measures were averaged to obtain a mean SPL value of the call.

### Call analysis

Patterns of inter individual variability of advertisement calls were assessed by analyzing calls from 14 males (10 calls per individual). From these calls we extracted the following parameters: sequence duration (Sd), call duration (Cd), duration of inter-call silences (Dics), pulse duration (Pd), number of pulses per call (Np/C), duration of inter pulse silences (Dips), call rate (Cr), rhythm (R, sum of signals/silences), pulse rate (Pr), dominant frequency (Df), frequency values of the first impulse (Ffi) and the last impulse (Fli), and frequency modulation (FM) obtained by subtracting Ffi from Fli. Frequency measurements were performed on power spectra with the following Fast Fourier Transform (FFT) values: Hamming window size  = 4096 points, filter bandwidth  = 120 Hz (T = 1/F =  8.3 ms). For each call parameter, we determined the mean, the standard deviation, the minimum and the maximum, Table 2. All these parameters were measured using Syntana [27].

### Ambient noise measurements

We measured the mean level of the ambient noise in two representative sites: along a riverbank and in the undergrowth of the rainforest. The sound level meter was positioned at a height of 0.5 m. Mean instantaneous sound pressure level measurements were taken at each site at 15 s intervals during 5 min for each individual encountered on different days (N = 10 measures). To compare the levels of the ambient noise at the two sites, we used non-parametric tests (Mann-Whitney U test). Note, however, that these measurements cannot fully quantify the entire variability at the two sites and should thus be regarded as a temporal & spatial snapshot of the ambient sound environment of this species.

### Propagation experiments

We broadcasted a representative advertisement call of the Harlequin frog. The call was repeated 15 times at intervals of 2.5 s at the natural sound pressure level of the species. The frequency and temporal values of this call and its repetition rate corresponded to the mean values measured in natural calls of the species. This series of repeated calls was broadcasted in the two natural habitats mentioned above. The propagation distances, i.e. the distances between the loudspeaker and the microphone, were the same in both experiments: 1 m (reference signal), 2 m (average distance between two territories of two neighbours males), 4 m, 8 m and 16 m. Experiments were conducted between 06.00 a.m. and 5.30 p.m. The loudspeaker and the microphone were placed 50 cm above the ground to mimic the natural position of calling males.

### Analysis of the propagated signal

The attenuation of the call during propagation and modification of the amplitude modulation of the signal before and after propagation through the environment (riverbank and undergrowth) were measured. The call attenuation, expressed in decibels, was defined as: CA  = 20 log (As/Ais), where As is the mean absolute amplitude value of the propagated call, and Ais is the mean absolute amplitude value of the reference signal. We compared the amplitude values of the signals recorded at 2 m, 4 m, 8 m and 16 m to the corresponding amplitude value of the reference signal recorded at 1 m. This allows us to compensate for the contribution of background noise to the amplitude measures. The attenuation values in the two environments were compared using non-parametric tests (Mann-Whitney U test). To analyse the modification of the amplitude modulation, the envelope of each call was calculated using the analytic signal calculation [33]. Each envelope (N = 3670 points) was digitally filtered using a short-term overlapping (80%) FFT (window size 4096, bandpass 0–250 Hz) focusing only on the slow amplitude modulation of the call. To minimize the influence of environmental changes the 15 envelopes corresponding to each test situation and distance of propagation were averaged. To assess the degree of similarity, a Bravais-Pearson correlation coefficient (r) between each of the 3670 points of the averaged envelope of the propagated signal and the corresponding points of the control signal was computed.

### Histology

Two specimens were killed by an overdosis of anaesthetic (ketamine) and fixed in either neutral-buffered formalin (4–10%) or ethanol, embedded in paraffin, sectioned at 5 µm and stained with standard Azan trichrome [34].

### Holotomography

One specimen were killed by an overdosis of anaesthetic (ketamine) and fixed in 3.7% formaldehyde solution and placed in a small polypropylene tube for holotomographic imaging [35]. Images were obtained using X-ray synchrotron radiation at the ID19 beam-line of the European Synchrotron Radiation Facility (ESRF, Grenoble). Images were taken in the phase contrast mode, with a pixel size of 7.46 and 10 µm at three (18, 83, 283, and 973 mm) and four (40 mm, 300 mm and 995 mm) sample-detector distances, respectively. The beam energy was set respectively at 20.5 and 17 keV. Twelve and nine hundred radiographic images (2048×2048 and 1024×1024 pixels) were acquired respectively using a FReLoN CCD Camera [36] at different angles ranging between 0 and 180°. Dark current and reference images without sample were recorded to perform flat field corrections on the projections. Phase retrieval was performed using the mixed approach described in [37]. Due to strong low-frequency noise in the resulting reconstructions, phase-absorption duality regularization [38] was incorporated, with the regularizing term set to correspond to bone (refractive index-absorption index ratio δ/β = 284). After phase retrieval, tomographic reconstruction was performed using a 3D version of the filtered back projection algorithm to reconstruct the 3D refractive index distribution. From this the 3D skull structure and soft tissue details was extracted.

### Microscopic MRI

The experiments were carried out at 9.4T on a vertical spectrometer (Inova,Varian) equipped with a 2T/m gradient coil and a home-built “loop-gap coil” (12 mm inner diameter). Three dimensional gradient echo experiments were performed with a repetition time of 84 ms and echo times of 2.4, 70 and 100 ms. The field of view was 1.6×1.1×1.1 cm^3^ and the acquisition matrix was 256×256×256 pixels, leading to a calculated resolution of 62.5×43×43 µm^3^. Three dimensional renderings were obtained after semi-automatic segmentation of the skeleton, using Avizo 6.1 (VSG, Visualization Sciences Group, Merignac, France) and the public domain program ImageJ (developed at the United States National Institutes of Health).

### Signals used in playback experiments

Signals were modified in the time and frequency domains using Syntana software as follows:

1. Suppression of amplitude modulation. The advertisement call of *Atelopus franciscus* is characterized by an increase in amplitude from the beginning to the end of the call. To test whether or not this amplitude variation is a useful parameter for species-specific recognition we removed all amplitude variations while keeping all the other acoustic parameters of the natural signal (Figs. 3, 7a). To do so we used the analytic signal calculation which allows amplitude de-modulation using the Hilbert transformation [33].

2. Inversion of the temporal structure. The advertisement call consists of a temporal succession of pulses with an inter-pulse period decreasing from the beginning to the end of the call. To test the importance of this temporal succession, we built a reversed call (Figs. 3d, 6).

3. Simplification of pulse syntax. The advertisement call typically comprises two parts: the first part corresponds to a succession of 20 pulses on average, emitted at a relatively slow rate and low intensity level, and the second part corresponds to a succession of 14 pulses on average, emitted at a relatively fast rate and high intensity level. To investigate which kind of pulse elicits territorial responses, we built two signals: one corresponding to the first part only and the other to the second part only (see Figs. 3a, 6).

4. Modifications of pulse and inter-pulse durations. The calls of *A. franciscus* show a distribution of pulse durations ranging from 2 to 6 ms with an inter-pulse duration ranging from 0.25 to 133.10 ms. To test the importance of these durations, we built 6 experimental signals. Using a synthesis method, we built pulses with the natural intra-pulse frequency but that were shortened or lengthened compared to the natural ones. As we chose to keep the natural pulse rate (number of pulses by unit of time), the result was a signal with the inter-pulse durations modified. Consequently, the tempo (sound/silence duration) was modified but the natural rhythm of pulse emission was kept. The decrease or increase of the pulse durations (in %) was as follows: −60, −50, −30, +30, +50 and 60 (Fig. 7).

5. Suppression of low frequency modulation (low FM). Some parts of the calls of *A. franciscus* are more or less modulated in frequency. To test the importance of the FM in species-specific recognition, a natural envelope was applied to a carrier frequency without FM. The amplitude envelope applied was extracted from a natural call, using the analytic signal calculation [33]. The carrier frequency represented the average frequency of a natural advertisement call (Figs. 3f, 6).

6. Shift of the frequency. According to our analysis, the calls of *A. francisus* present a distribution of frequencies between 2700 and 3700 Hz. To estimate if the species-specific coding process is based on the precise frequency values of the signal, a natural call was shifted up in frequency. This was done by picking a data record through a square window, applying short-term overlapping (50%) FFT, followed by a linear positive shift of each spectrum, and by a short-term inverse FFT [39]. The linear shifts of the spectra were: +200, +400, +800, +1200 and 2000 Hz.

### Playback design

Prior to the start of a test, we positioned the speaker at a distance of 2.5 m from the subject, which is the typical distance between adjacent territorial males [6]. We chose to test males that had no neighbours within a 10 m diameter range to avoid interactions with other territorial males. Males were stimulated by 2 series of signals separated by 2 minutes of silence. One series, the control series, consisted of 15 consecutive advertisement calls (with 34 pulses on average) separated by natural inter-call silences (mean duration: 2.5 s). The second series, being the experimental one, consisted of 15 experimental calls (a modified natural call or a synthetic signal repeated) separated also by natural inter-call silences. The order of presentation of both control and experimental series was randomised. In natural conditions, the response to an advertisement call was characterised by an obvious behavioural change in the male's attitude: it turned its body in the direction of the sound source, called in reply (by an advertisement and/or a territorial call), and then approached the loudspeaker. The intensity of responses of tested males to playback signals was evaluated by a five-point scale ranked as follows: class 0 (none)  =  no reaction; class 1 (weak)  =  orientation towards the loudspeaker; class 2 (medium)  =  orientation + approach towards the loudspeaker + emission of an advertisement call (Audio S1); class 3 (strong)  =  orientation + approach + emission of a territorial call (Audio S2); class 4 (very strong)  =  similar to class 3 but territorial call emission after the first signal, fast approach in the direction of the loudspeaker and stop in the vicinity (less than 2 m). This behavioural scale is similar to the one used in a previous study dealing with a related species of Harlequin frog [5].

### Data analysis

The sign test [40] was used to compare the responses to experimental signals and to the control signal. One-tailed sign tests were computed using Statistica Version 1.5 at alpha  = 0.05.

## Supporting Information

Audio S1 **Recording of a male advertisement call.**
(WAV)

Audio S2 **Recording of a male territorial call.**
(WAV)

Video S1 **3D reconstruction of the ear based on synchrotron microtomography.**
(AVI)

Video S2 **3D animation illustrating the anatomy of the head as revealed by microscopic MRI.**
(AVI)
